# DLC-Coated Ferroelectric Membranes as Vascular Patches: Physico-Chemical Properties and Biocompatibility

**DOI:** 10.3390/membranes11090690

**Published:** 2021-09-07

**Authors:** Yuri Yuriev, Semen Goreninskii, Artem Runts, Elisaveta Prosetskaya, Evgenii Plotnikov, Darya Shishkova, Yulia Kudryavtseva, Evgeny Bolbasov

**Affiliations:** 1 B.P. Veinberg Research and Educational Centre, Tomsk Polytechnic University, 634050 Tomsk, Russia; yurjev@tpu.ru (Y.Y.); artemshift@tpu.ru (A.R.); eap47@tpu.ru (E.P.); 2 Microwave Photonics Laboratory, V.E. Zuev Institute of Atmospheric Optics SB RAS, 634055 Tomsk, Russia; 3 N.M. Kizhner Research and Educational Centre, Tomsk Polytechnic University, 634050 Tomsk, Russia; sig1@tpu.ru; 4 Research School of Chemistry & Applied Biomedical Sciences, Tomsk Polytechnic University, 634050 Tomsk, Russia; plotnikovev@tpu.ru; 5 Research Institute for Complex Issues of Cardiovascular Diseases, 650002 Kemerovo, Russia; Shishkova@cardio.kem.ru (D.S.); kudrua@cardio.kem.ru (Y.K.)

**Keywords:** ferroelectric membranes, DLC coatings, vascular patches

## Abstract

In this paper, the results on the fabrication of ferroelectric membranes as vascular patches with modified surfaces are presented. For the modification of a membrane surface contacting blood, DLC coating was deposited using the pulsed vacuum arc deposition technique. The physico-chemical properties and cytotoxicity of the membranes modified under various conditions were studied. It was found that DLC coatings do not affect membrane microstructure, preserving its crystal structure as well as its high strength and elongation. It was revealed that an increase in the capacitor storage voltage results in the rise in sp^2^- and sp-hybridized carbon concentration, which makes it possible to control the chemical structure and surface energy of the modified surface. The experiments with 3T3L1 fibroblasts showed no toxic effects of the materials extracts.

## 1. Introduction

According to the World Health Organization, cardiovascular disease (particularly atherosclerosis (stenosis) of the carotid artery) remains one of the main causes of mortality in developed and developing countries [1,2]. The outbreak of the COVID-19 infection makes the problem even more relevant [3]. One of the main approaches for the restoration of carotid artery permeability is reconstruction with vascular patches after surgical thrombus removal [4]. Autologous veins remain the gold standard of vascular patches, but their deficiency significantly limits the availability of such medical help [5]. For that reason, researchers around the world are looking for novel materials for effective carotid artery restoration.

Owing to their high strength, non-toxicity, chemical stability, and hemocompatibility, electrospun fibrous polymer membranes made of polyvinylidene fluoride and its copolymers, trifluoroethylene (VDF-TrFE) and tetrafluoroethylene (VDF-TeFE), appear to be promising materials for the development of vascular patches [6]. The ferro- and piezoelectric properties of such membranes enhance the adhesion and proliferation of various cells, thus improving vascular patch endothelialization and decreasing the possibility of post-surgical complications [7,8,9].

One of the key problems with membranes made of PVDF and its copolymers is their thrombogenicity. In order to overcome this drawback, various strategies for the modification of membranes resulting in the hindrance of protein adsorption have been proposed. Among the common modification approaches are: the formation of organic coatings [10], ion irradiation [11], polydopamine-mediated atom transfer radical polymerization [12], and composite membrane fabrication [13].

The fabrication of diamond-like carbon (DLC) coatings on the membrane surface appears to be a promising approach for the reduction in vascular patch thrombogenicity induced by PVDF and its copolymers. Owing to the properties of DLC coatings, such as high hardness, low frictional coefficient, high wear and corrosion resistance, chemical inertness, high electrical resistivity, antibacterial activity, and high hemo- and biocompatibility, they can be applied to the surface of blood-contacting devices (vascular grafts, coronary stents, heart valves, catheters, hemodialyzers, and heart-lung bypass systems) in order to lower their thrombogenicity [14,15,16,17]. Thus, DLC coatings formed on the surface of blood-contacting polymer ferroelectric vascular patches may reduce their thrombogenicity. The further modification of DLC coatings by physical and chemical approaches imparts them with antithrombogenic properties, and stimulates the adhesion and proliferation of various cell types [18,19]. Thus, DLC coatings may be applied in order to decrease the thrombogenicity of the inner surface of the vascular patch, as well as for the enhanced endothelialization of its outer surface.

The following techniques are used for the deposition of DLC coatings on metal and ceramic implants: magnetron sputtering, ion beam deposition, vacuum-arc evaporation, pulsed laser deposition, mass selected ion beam deposition, and plasma-based ion implantation [20,21]. The listed methods of DLC coating fabrication require accelerating potential to the substrate, resulting in a significant rise in the substrate temperature, or they do not have a sufficient deposition rate. These factors limit the applicability of the listed techniques for the deposition of coatings on the surface of ultrathin fibrous polymer membranes.

The pulsed vacuum arc deposition method appears to be a promising approach for the fabrication of DLC coatings on the surface of polymer membranes [22,23]. This method allows for the generation of carbon plasma with ion energy in the range of 40–90 eV by changing the capacitor storage voltage, does not require accelerating potential to the substrate, provides a high rate of condensate formation (up to 1 × 10^4^ Å/s), and the substrate temperature does not exceed 70 °C [24]. These features make the pulsed vacuum arc deposition method suitable for the deposition of DLC coatings on the surface of vascular patches made of PVDF and its copolymers. However, until now, there have been no reports on the application of DLC coatings on the surface of vascular patches made of PVDF and/or its copolymers. The physico-chemical and biomedical properties of the modified membranes and the DLC coatings formed on their surface have not been reported. This is limiting the application of DLC coatings for the modification of vascular patches, hindering the utilization of these membranes in clinical practice, and restricting the application of pulsed vacuum arc deposition as a multifunctional method for the treatment of various surfaces (metals, ceramics, and polymers). In the present work, we report the possibility of DLC coating deposition on the surface of ferroelectric polymer membranes by means of pulsed vacuum arc deposition, as well as the physico-chemical properties and biocompatibility of the modified membranes, for the first time.

## 2. Materials and Methods

### 2.1. Electrospinning of VDF-TeFE Membranes

The membranes were prepared from a 6% wt. solution of VDF-TeFE copolymer (VDF/TeFE ratio 80/20, Halopolymer, Moscow, Russia) in acetone. Membrane fabrication was conducted using the electrospinning technique (NANON-01A, MECC CO., LTD, Fukuoka, Japan) on an aluminum cylindrical collector with a 200 mm length and a 100 mm diameter. The distance between the injector (24G needle) and the assembly manifold was 90 mm. The voltage of the injector was 25 kW. The flow rate of the spinning solution was 4 mL/h, and the assembly speed was 200 rpm. To remove the residual solvents, the fabricated membranes were stored in a VD 115 vacuum furnace (Binder, Tuttlingen, Germany) at a temperature of 100 °C and a 0.1 Pa pressure for 24 h.

### 2.2. Deposition of DLC Coatings by the Pulsed Vacuum Arc Deposition Technique

Prior to the DLC coating formation, the membranes were placed on a metal substrate fixed in a special holder that provides rotation around itself and the setup perimeter for the formation of a uniform coating (Figure S1). The formation of DLC coatings on the membrane surfaces was conducted by the pulsed vacuum arc deposition method with the sputtering of a high-purity graphite (99.99%) cathode. The following parameters were applied during the coating deposition: a striking voltage of 400 V, a pulse frequency of 3 Hz, 3000 pulses per sample, and a minimum target-to-membrane distance of 250 mm. The pulsed arc was powered by a 2000 μF capacitor bank. Four sample groups were formed with respect to the used capacitor storage voltage (150, 200, 250, and 300 V). Non-modified samples were used as a control group. Before the DLC coating deposition, the samples were cleaned with argon ions. An ion source with Hall electron drift was used for cleaning with the following parameters: an ion source voltage of 1 kV, a discharge current of 30 mA, a chamber pressure of 2.1 × 10^−2^ Pa, and a treatment time of 40 s.

### 2.3. Membrane Characterization

#### 2.3.1. Scanning Electron Microscopy (SEM)

The morphology of the membranes was investigated by scanning electron microscopy (SEM) (VEGA 3 SBH, Tescan, Warrendale, PA, USA). The fiber diameter of the membranes was determined from the SEM images out of 10 different fields of view using Image J 1.38 software (National Institutes of Health, Bethesda, MD, USA). To calculate an average diameter, at least 350 fibers were measured.

#### 2.3.2. Tensile Strength and Relative Elongation

The tensile strength and the relative elongation of the samples were investigated according to the recommendations of ISO 9073.3:1989 using the Instron 3344 tensile testing machine (Instron, Norwood, MA, USA) with a sample preload of 0.1 N and a crosshead speed of 10 mm/min.

#### 2.3.3. X-ray Diffraction Analysis (XRD)

The crystal structure of the membranes was investigated using the X-ray diffraction method with an XRD 6000 diffractometer (Shimadzu, Kyoto, Japan). The average size of the crystallites (*l_c_*) was calculated using the Debye-Scherrer Equation (1):

(1) $$l_{c}=\frac{k\lambda }{Cos\theta \beta }$$

where *λ* is the wavelength of the incident radiation (Cu K-alpha, *λ* = 1.54056 Å), *β* is the width of the reflection at a half height (FWHM), *θ* is the angle of the diffraction, and *k* = 0.9. The estimation of the FWHM parameter was performed by interpolating the X-ray pattern using a Gauss function (Origin 2021, Origin Lab., Northampton, MA, USA).

#### 2.3.4. Raman Spectroscopy

Raman spectra were taken using an InVia spectrometer (Renishaw, Gloucester, UK) equipped with a DM 2500 M microscope (Leica, Wetzlar, Germany) with a 50X objective. Lasers with the power of 100 mW, wavelengths of 532 nm, and a spectral resolution of 2 cm^−1^ were used. A spectral range of 500–2500 cm^−1^ was considered. The Raman spectra were deconvoluted using Gaussian line fitting (Origin 2021, Origin Lab., Northampton, MA, USA). The fitting parameters were used to calculate the Raman parameters, including band position and the spectral intensity ratios I_D_/I_G_ and I_max_/I_C_.

#### 2.3.5. Wettability and Surface Energy

The samples’ wettability was characterized by depositing 3 μL drops of polar (Milli-Q water (H_2_O)) and non-polar (diiodomethane (CH_2_I_2_)) liquids using an Easy Drop (Easy Drop, KRÜSS GmbH, Hamburg, Germany) contact angle measurement system. Droplets were placed at different positions on samples and images were captured after a 2 min deposition of each drop. According to the results of the contact angle measurement, surface free energy (σ) and its dispersive (σ^D^) and polar (σ^P^) components were calculated using the Owens–Wendt–Rabel–Kaelble (OWRK) method.

#### 2.3.6. Cytotoxicity of Membrane Extracts

The study of the interaction of the obtained membranes with cells in vitro was carried out using mouse embryonic fibroblasts 3T3L1. Sterile membrane samples with a diameter of 12 mm were placed into the wells of 24-well plates and filled by 100 μL cell growth media per well. The samples were left for 5 days in a CO_2_ incubator at 37 °C for extraction. For all cell experiments, we used the same media, DMEM (Gibco, Gaithersburg, MD, USA), supplemented with the glutamine supplement GlutaMAX (Gibco, Gaithersburg, MD, USA), 10% fetal bovine serum One Shot^®^, (Thermo Fisher Scientific, São Paulo, Brazil), and antibiotics (penicillin/streptomycin mixture) (Paneko, Moscow, Russia). After extraction, media were used for cell growth. A medium without polymer disks was used as a control and kept in the same conditions for 5 days. The cells were cultured for 24, 72, and 168 h in an atmosphere containing 5% CO_2_ at a temperature of 37 °C. After incubation, media from each sample were used for cytotoxicity and cell growth in the MTT assay. To perform the MTT test, the medium in the plate was replaced with a solution of 3-(4,5-dimethylthiazol-2-yl) -2,5-diphenyl-2H-tetrazolium bromide (MTT) at a concentration of 0.45 mg/mL, and the plate was placed in a thermostat for 4 h. The MTT solution was then removed and DMSO was added to dissolve the formazan. Next, the optical density was measured at a wavelength of 570 nm, and the viability calculation was performed and presented as a percentage of the control group.

The absolute number of cells per 1 mm^2^ of the surface was evaluated using a fluorescence microscopy system (AxioVert.A1, Carl Zeiss, Oberkochen, Germany). Cells were stained with vital fluorescent dyes Calcein AM 0.5 μg/mL (Abcam, Cambridge, MA, USA) for the green staining of the cells’ cytoplasm, and Hoechst 33342 1 μg/mL (Sigma-Aldrich, St. Louis, MI, USA) for the blue staining of the nuclei of all adhered cells. The dyes were applied to the samples 15 min before the microscopy. Image processing was performed using ZEN pro software (Carl Zeiss, Oberkochen, Germany). Cells were counted using ImageJ 1.38 software (National Institutes of Health, Bethesda, MD, USA) from 10 different fields of view. The studies were performed on 5 samples of each studied group in triplicate using 10 randomly selected fields of view for each group. Cells cultured in media without material extraction were used as a control.

#### 2.3.7. Statistical Analysis

Statistical analysis was performed in GraphPad Prism 8.00 (GraphPad Software, La Jolla, CA, USA). The data are shown as mean (SD) ± standard deviation (SD).

## 3. Results and Discussion

### 3.1. DLC Coatings Deposition

**Figure 1:**
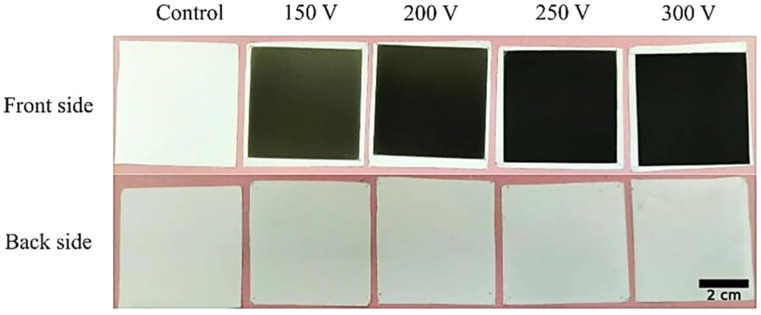
The photographs of the front and back sides of the membranes after the deposition of DLC coating under various capacitor storage voltages.

The photographs of the front and back sides of the membranes before and after the deposition of the DLC coating under various capacitor storage voltages are presented in Figure 1. The front and back sides of the original polymer membrane were not different from each other and had a uniform white color. Coating deposition resulted in the change of the front side color from brownish (150 V) to black (300 V). Macro defects such as burns and melting were not observed. The samples’ color was uniform. The color of the back side of the sample was not changed under all the tested deposition parameters. Thus, single-side modification of the membrane is possible.

### 3.2. Scanning Electron Microscopy (SEM)

**Figure 2:**
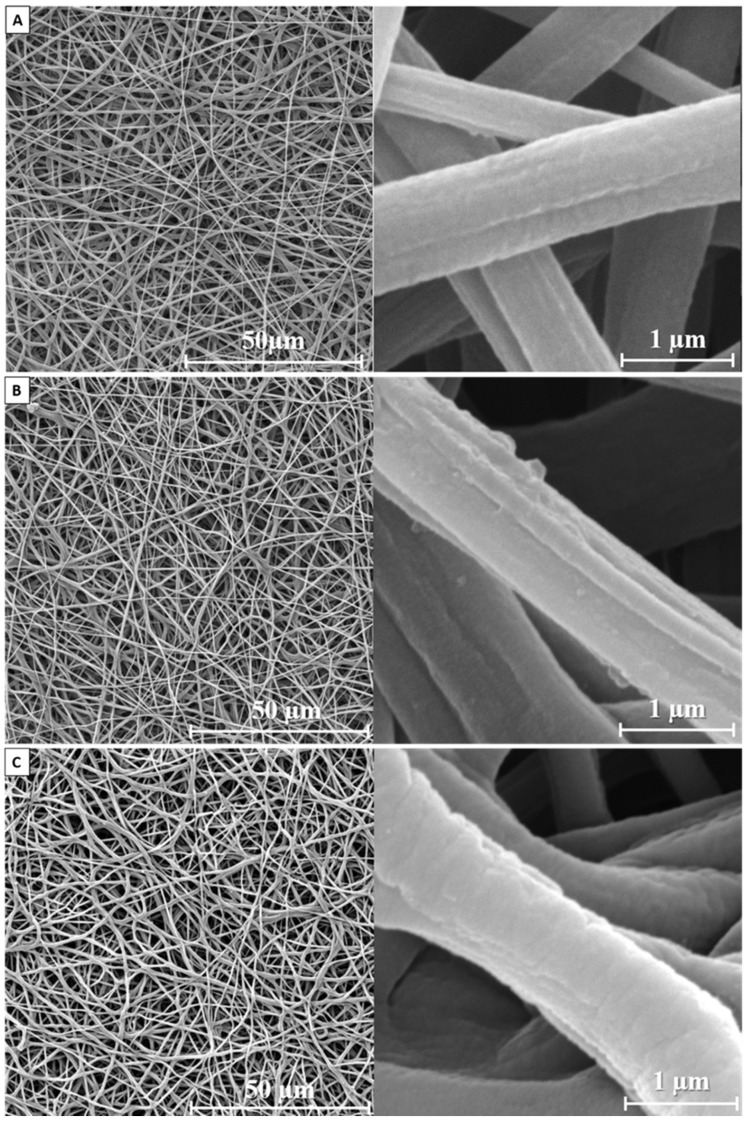
SEM images of the front membrane surface: (**A**)—non-treated, (**B**)—150 V, (**C**)—300 V.

SEM images of the membrane surface before and after DLC coating deposition are presented in Figure 2. Control membranes were formed by chaotically interwoven cylindrical fibers. Fiber defects (such as beading and thickening) were not observed. At higher magnifications, the fibers’ microrelief may be observed. It is presented by cavities directed along the fiber and uniformly distributed along the fiber surface. Average fiber diameter was found to be 0.61 ± 0.21 µm. Coating deposition under the tested parameters did not result in changes to the membrane structure. The fibers remained sustained and defects were not observed.

An increase in the capacitor storage voltage resulted in changes in the fibers’ surface relief. At 150 V, a number of cavities were observed on the smooth fibers’ surfaces (Figure 2b). At 300 V, these cavities were filled and the fiber diameter increased slightly (Table 1). Thus, the presented results demonstrate the highest coating deposition rate within a voltage range of 250–300 V.

### 3.3. Tensile Strength and Relative Elongation

The ultimate tensile strength and relative elongation of the intact membrane under longitudinal strain were found at 17.8 ± 1.8 MPa and 65.3 ± 9.0%, respectively. DLC coating formation resulted in a decrease in the membrane strength and elongation in all studied groups. The maximum decrease was observed for the samples coated under capacitor storage voltages of 250 and 300 V (Table 1). A decrease in the membranes’ strength and relative elongation with the voltage increase may be explained as follows: A voltage increase in the range of 100–400 V results in a decrease in the energy of carbon ions from 30 to 90 eV [25]. The interaction of high-energy ions with polymer fibers leads to the formation of surface defects. The sizes and amount of the formed defects are proportional to the ion energy [26]. As the strength of ultrathin fibers is significantly affected by their surface properties [27], it may be proposed that the formation of defects on the front side of the membranes significantly reduces their strength and elongation.

### 3.4. X-ray Diffraction Analysis

**Figure 3:**
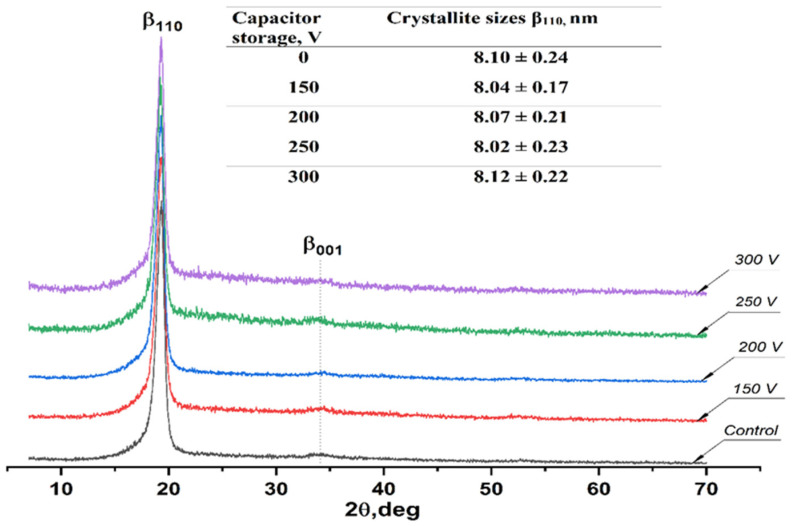
X-ray diffraction patterns of the obtained membranes.

X-ray diffraction patterns of the untreated membranes and the samples with deposited DLC coating are presented in Figure 3. The XRD pattern of the pristine VDF-TeFE membrane exhibits an intense peak at 19.3°, corresponding to the (110) crystallographic plane, and a broad peak at 34.6°, corresponding to the (001) crystallographic plane of the electrically active β phase of the VDF-TeFE copolymer [28]. The presence of the β phase indicates ferroelectric and piezoelectric properties in the produced polymer membranes [29]. The formation of electrically active crystalline phases in electrospun non-woven materials based on polyvinylidene fluoride and its copolymers is caused by the polarization of the polymer under the influence of a high-intensity electric field in the space between the needle and the collector [30]. Unlike the DLC coatings deposited using the reactive magnetron sputtering method, the coatings obtained using the pulsed vacuum arc deposition technique were found to be amorphous regardless of the capacitor storage voltage. This finding is confirmed by the absence of reflexes at 43.2°, 44.3°, and 50.4°, corresponding to diamond, carbon, and graphite, respectively [31]. The fabrication of DLC coatings under the tested parameters had no effect on the crystal structure of the VDF-TeFE membranes, which is evidenced by the absence of statistically significant changes in β phase crystallite sizes in the (110) crystallographic plane. The absence of β phase reflexes proves the preservation of interplanar spacing (Figure 3).

Thus, it was found that during the DLC coating deposition, polymer membranes preserve their ferroelectric and piezoelectric crystal structure. This finding may be useful for drug delivery and the active stimulation of membrane–native tissue integration [32,33].

### 3.5. Raman Spectroscopy

**Figure 4:**
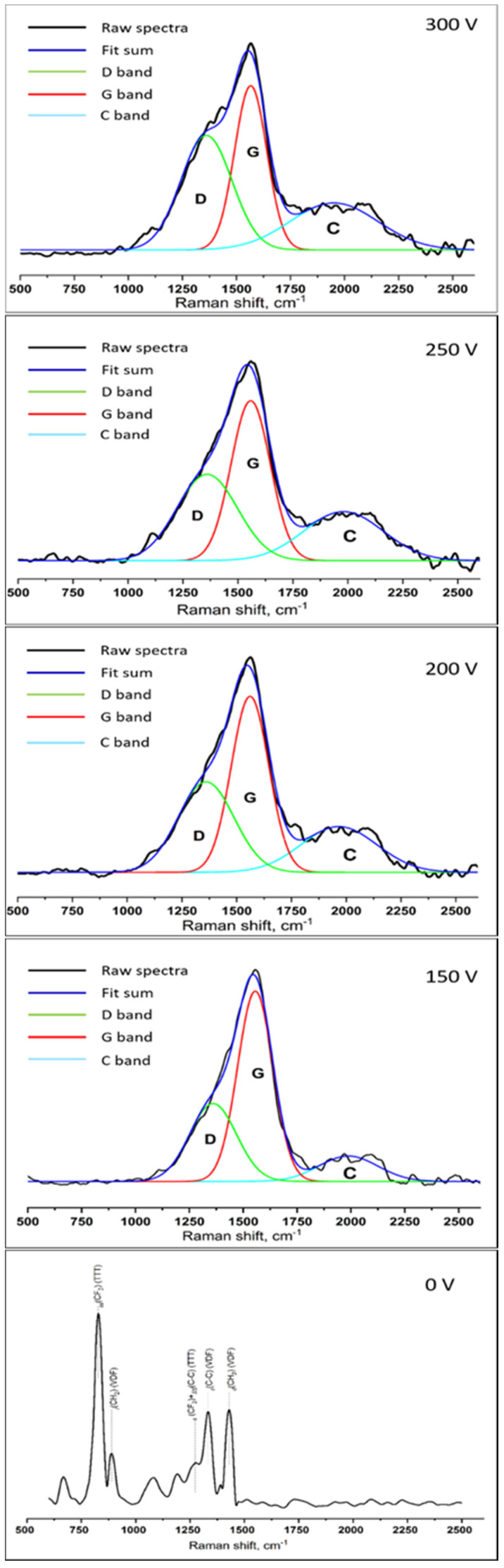
Raman spectra of the obtained membranes.

The Raman spectra of the obtained materials are presented in Figure 4. The spectrum of the initial polymer membrane presents a number of intensive bands. A strong Raman band at 834 cm^−1^ is assigned to the symmetric stretching mode of the CF_2_ groups in the all-trans (TTT) conformation. The band at 885 cm^−1^ is characteristic of the VDF monomeric unit, which is assigned to the CH_2_ rocking mode. The Raman band at 1280 cm^−1^ (a coupling of CF_2_ stretching and skeletal C–C stretching modes) is also characteristic of the all-trans conformation. The band at 1430 cm^−1^ is assigned to the –CH2 deformation mode characteristic of the VDF sequence. The band at 1330 cm^−1^ is assigned to the C–C stretching mode characteristic of the VDF sequence [34,35,36]. The absence of a Raman band at 810 cm^−1^, attributed to the gauche form, confirms our conclusions on polymer β phase crystallization based on XRD results. Two wide bands may be observed on the spectra of the samples with DLC coatings. The first one is an intensive band in the range of 1000–1750 cm^−1^ corresponding to the G and D peaks’ (1560 and 1360 cm^−1^, respectively) superposition. The G and D peaks are due to sp^2^ bonded carbon atoms. The G peak is due to the bond stretching of all pairs of sp^2^ atoms in both rings and chains. The D peak is due to the “breathing” modes of sp^2^ atoms in rings, or to an sp^3^ network structure. That wide peak is typical for amorphous carbon structures [37,38]. The second, less intensive band lies in the range of 1820–2200 cm^−1^ and corresponds to the linear polymeric sp-hybridized carbon chain (carbynes), either in the form of cumulenes (C=C)n, or as polyynes (C≡C)n, which are known to have Raman bands in the spectral range associated with the stretching vibrations of C=C bonds [39,40]. With the increase in the capacitor storage voltage, the G peak shifted toward higher wavenumbers, an increase in the intensity of the D peak, and the I_D_/I_G_ ratio was observed (Figure 4, Table 2).

These changes in Raman spectra are caused by the clustering of atoms having sp^2^ hybridization. Therefore, the increase in the capacitor storage voltage favors the rearrangement of the amorphous matrix, which leads to an increased number of closed carbon rings in sp^2^ clusters and/or the number of clusters containing rings [24]. The decrease in the I_max_/I_c_ ratio with the voltage rise (Table 2) is most likely due to the increase in the length of cumulene-like chains (=C=C=) and/or the formation of polyyne-like chains (–C≡C–), and may occur as a result of DLC crystal structure rearrangement under the greater energy of carbon plasma ions [24]. Thus, the conducted studies demonstrate the possibility of DLC coating composition control accompanied by the preservation of the membrane structure.

### 3.6. Wettability and Surface Energy

The water and diiodomethane contact angles of the obtained membranes, as well as the surface energy (σ) and its dispersive (σ^D^) and polar (σ^P^) components, were calculated using the OWRK method and are presented in Table 3. Non-modified membranes demonstrate extremely low surface free energy (Table 3), which is due to the low electronegativity of fluorine and the high porosity and heterogeneity of the membrane surface [41,42]. The DLC coatings deposited on the membrane surface had no effect on the water contact angle. The diiodomethane contact angle decreased 75% and 88% in the case of coatings deposited under 150 and 300 V, respectively (Table 3). The decrease in the non-polar liquid contact angle evidences an increase in the DLC-coated membranes’ surface free energy of more than 10 times compared to the control (Table 3).

The rise in free surface energy from its dispersive component was described by Kalin et al. [43] in the case of poly(tetrafluoroethylene). The increase in free surface energy after the deposition of the DLC coating may be due to the combined effects of non-polar interaction (or dispersive component) induced by sp^2^ bonds, and polar interaction by dangling bonds [44].

### 3.7. Cytotoxicity of Membrane Extracts

**Figure 5:**
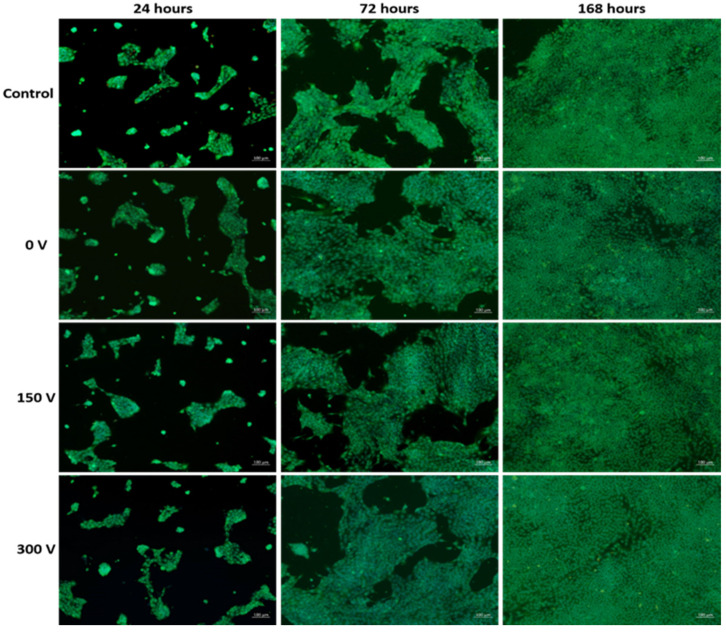
Images of the fluorescently labeled fibroblasts after 24, 72 and 120 h of cultivation with sample extracts.

Micrographs of the cells cultured in the wells of 96-well plates with 5 day extracts of the obtained membranes with DLC coatings deposited under various conditions are presented in Figure 5.

After 24 h of cultivation, fusiform fibroblasts with well-defined round or oval nuclei were observed in all studied groups. The cells were conglomerated in clusters with the square up to 0.03 mm^2^. Cell viability parameters were not significantly different between the studied groups (Table 4). After 72 h of cultivation, the cells remained fusiform and highly viable. This is evidenced by the 2.5-fold increase in the cell density in comparison with 24 h of cultivation (Table 4). After 168 h of cultivation, a confluent fibroblast monolayer was formed in all studied groups (Figure 5). After 168 h, viability characteristics were not significantly different compared to the control.

The slight decrease in cell viability after 168 h of cultivation, compared to a 24-h period, is the result of culture medium depletion and the accumulation of cell waste products. Thus, the conducted studies demonstrate that, regardless of the parameters for DLC coating deposition, the modified membranes have high biocompatibility and contain no toxic compounds, which are able to suppress 3T3L1 fibroblast growth.

## 4. Conclusions

The possibility of DLC coating deposition on the surface of ferroelectric polymer membranes by means of pulsed vacuum arc deposition was demonstrated. The obtained materials are promising for the development of vascular patches. It was shown that DLC coatings formed on the front side of the membrane do not change its macrostructure, preserving its high strength and elongation, and are an efficient tool for surface modification. DLC coatings formed under the studied parameters were found to be amorphous. The process of DLC coating deposition had no effect on the membranes’ crystal structure, thus preserving its ferroelectric properties. It was revealed that with the increase in capacitor storage voltage, the concentration of sp^2^ and sp-hybridized carbon in the coating rises. Thus, it is possible to vary the chemical composition and surface energy of the membrane. Extracts of the obtained membranes were found to be non-toxic to 3T3L1 fibroblasts and did not affect cell proliferation.

## Figures and Tables

**Table 1:** Average fiber diameter and mechanical characteristics of the membranes modified under various conditions.

Capacitor Storage Voltage, V	Mean Fiber Diameter, µm	Tensile Strength, MPa	Elongation, %
0	0.61 ± 0.21	17.8 ± 1.8	65.3 ± 9.0
150	0.57 ± 0.24	15.7 ± 0.7 *	63.0 ± 4.7
200	0.56 ± 0.21	14.8 ± 0.5 *	61.7 ± 3.6
250	0.63 ± 0.22	14.0 ± 0.4 *	56.6 ± 2.6 *
300	0.67 ± 0.19 *	14.3 ± 1.1 *	42.2 ± 1.9 *

*, *p* < 0.05 compared to control (Kruskal–Wallis test).

**Table 2:** Parameters of Raman spectra of the obtained materials.

Voltage, V	G-Peak Position, cm^−1^	I_D_/I_G_	I_max_/I_C_
150	1556	0.46 ± 0.11	7.24 ± 0.58
200	1561	0.50 ± 0.14	4.32 ± 0.32 *
250	1558	0.55 ± 0.12 *	4.19 ± 0.28 *
300	1566	0.70 ± 0.18 *	4.35 ± 0.36 *

*, *p* < 0.05 compared to the control (Kruskal–Wallis test).

**Table 3:** Contact angles and free surface energy of the membranes with DLC coatings deposited under various parameters.

Voltage, V	Contact Angle, Degrees		Surface Free Energy, mJ/m^2^		
	H_2_O	CH_2_I_2_	σ	σ^D^	σ^P^
0	130.6 ± 2.0	109.1 ± 2.2	5.8 ± 0.2	5.7 ± 0.2	0.06 ± 0.02
150	126.0 ± 1.7	24.2 ± 3.4 *	66.3 ± 1.4	58.0 ± 1.1	8.3 ± 0.3
200	126.1 ± 1.8	12.4 ± 1.8 *	71.8 ± 0.8	62.4± 0.6	9.4 ± 0.2
250	127.1 ± 0.7	11.2 ± 2.0 *	73.0 ± 0.8	63.0 ± 0.7	10.0 ± 0.3
300	128.1 ± 0.9	12.6 ± 2.0 *	73.4 ± 0.9	63.0 ± 0.7	10.5 ± 0.2

*, *p* < 0.05 compared to the control (Kruskal–Wallis test).

**Table 4:** Results of testing the cytotoxicity of obtained membranes after 24, 72, and 168 h of cultivation.

Voltage, V	Number of Cells, mm^−2^	Cell Viability, %	Number of Cells, mm^−2^	Cell Viability, %	Number of Cells, mm^−2^	Cell Viability, %
	24 h		72 h		168 h	
0	225 ± 19	103 ± 5	615 ± 61	100 ± 8	924 ± 74	98 ± 9
150	222 ± 18	106 ± 8	631 ± 48	109 ± 7	977 ± 54	97 ± 6
200	203 ± 14	106 ± 6	634 ± 57	105 ± 9	953 ± 63	99 ± 10
250	223 ± 17	105 ± 8	663 ± 44	96 ± 10	989 ± 77	101 ± 6
300	208 ± 22	99 ± 10	655 ± 52	94 ± 12	932 ± 68	103 ± 5
Control	216 ± 31	100 ± 5	636 ± 49	100 ± 6	932 ± 61	99 ± 4

## Data Availability

Not applicable.

## Supplementary Materials

The following are available online at https://www.mdpi.com/article/10.3390/membranes11090690/s1, Figure S1: Layout of membrane samples in the chamber of the pulsed vacuum arc deposition.
