# Supplementary material for: DLC-Coated Ferroelectric Membranes as Vascular Patches: Physico-Chemical Properties and Biocompatibility
Source: Membranes (Basel). 2021 Sep 7;11(9):690. doi: 10.3390/membranes11090690 (PMC8470059; doi:10.3390/membranes11090690)
Supplement: Supplementary file 1 [file membranes-11-00690-s001.zip › membranes-1362811-supplementary.pdf]

membranes

Supplementary Material

DLC-Coated Ferroelectric Membranes as Vascular Patches: Physico-Chemical Properties and Biocompatibility

Yuri Yuriev [1,2], Semen Goreninskii [3], Artem Runts [1], Elisaveta Prosetskaya [1], Evgenii Plotnikov [4], Darya Shishkova [5], Yulia Kudryavtseva [5], Evgeny Bolbasov [1,2,*]

1 B.P. Veinberg Research and Educational Centre, Tomsk Polytechnic University, 634050 Tomsk, Russia. yurjev@tpu.ru (Y.N.); artemshift@tpu.ru (A.A.R.); eap47@tpu.ru (A.A.P.)
2 Microwave Photonics Lab, V.E. Zuev Institute of Atmospheric Optics SB RAS, 634055 Tomsk, Russia.

3 N.M. Kizhner Research and Educational Centre, Tomsk Polytechnic University, 634050 Tomsk, Russia.
4 Research School of Chemistry & Applied Biomedical Sciences, Tomsk Polytechnic University, 634050 Tomsk, Russia.
5 Research Institute for Complex Issues of Cardiovascular Diseases, 650002 Kemerovo, Russia.; Shishkova@cardio.kem.ru (D.S.); kudrua@cardio.kem.ru (Y.K.)
* Correspondence: Ebolbasov@gmail.com

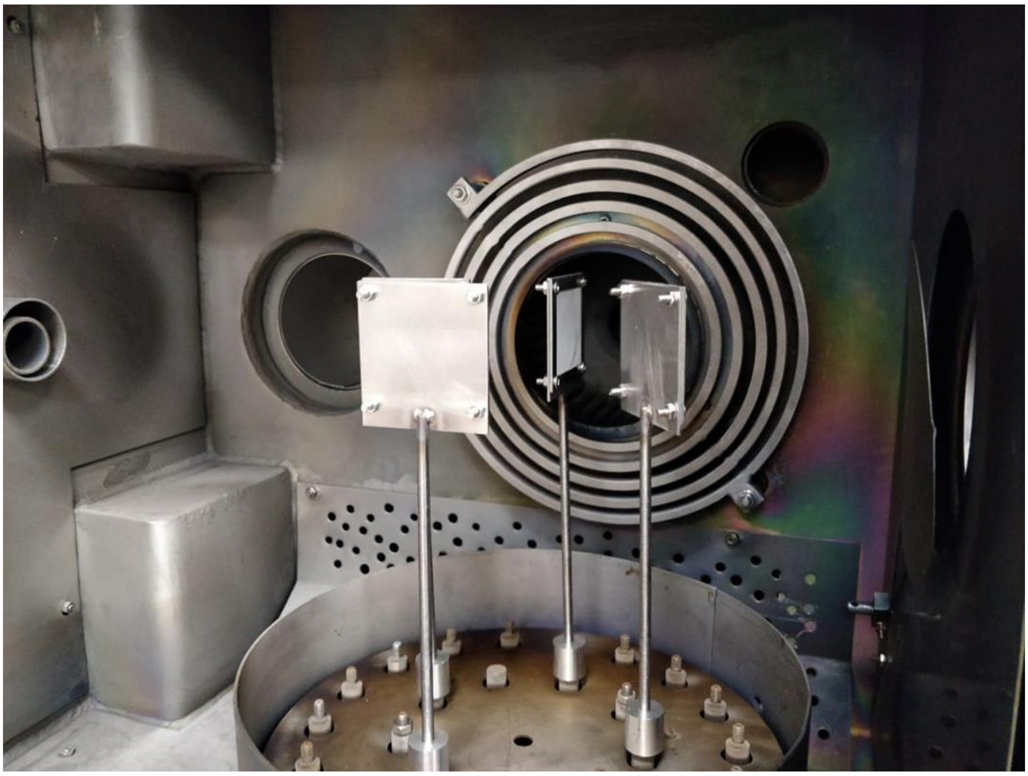

Figure S1. Installation for the formation of DLC coatings.
